# Total Leukocyte Count Depicting the Degree of Inflammation in Acute Appendicitis

**DOI:** 10.7759/cureus.17566

**Published:** 2021-08-30

**Authors:** Muhammad Bilal, Atif Yusufzai, Neelam Asghar, Ahmed Sohail, Zoobia Z Khan, Tehreem Zahid, Hassan Mumtaz, Shahzaib Ahmad

**Affiliations:** 1 Medicine, Benazir Bhutto Hospital, Rawalpindi, PAK; 2 Internal Medicine, Islamabad Medical and Dental College, Islamabad, PAK; 3 Clinical Research Center, Shifa International Hospital, Islamabad, PAK; 4 Research Center, Shifa International Hospital, Islamabad, PAK; 5 Medicine, Shifa International Hospital, Islamabad, PAK; 6 Clinical Research, Shifa International Hospital, Islamabad, PAK; 7 Critical Care Medicine-COVID ICU, KRL Hospital, Islamabad, PAK; 8 Surgery: Urology/Orthopeadics, KRL Hospital, Islamabad, PAK; 9 Internal Medicine - Paediatrics, Holy Family Hospital, Rawalpindi, PAK; 10 General Medicine, Surrey Docks Health Center, London, GBR; 11 Urology, Guys and St Thomas Hospital, London, GBR; 12 Physiology, Mayo Hospital, Lahore, PAK

**Keywords:** acute appendicitis, normal total leukocyte count, histopathology, inflammation, quality of life (qol)

## Abstract

Introduction

Acute appendicitis (AA) is the most common cause of acute abdomen in young adults. The diagnosis is usually made on various clinical findings. However, a missed case of acute appendicitis is a catastrophe as it later presents with life-threatening complications and results in medicolegal issues. Raised total leukocyte count (TLC) is a frequent finding in patients with acute appendicitis. As a convention, a normal leukocyte count usually rules out the differential diagnosis of acute appendicitis. Recent studies claimed that a substantial proportion of patients with normal TLC also had acute appendicitis and warranted a careful evaluation of such cases before sending them home. However, the reported frequency of acute appendicitis among TLC normal patients varied greatly among studies which necessitated the present study.

Aim

Our aim was to determine the frequency of acute appendicitis in patients of normal TLC.

Materials and methods

This descriptive cross-sectional study was conducted at the Department of Surgery, KRL Hospital Islamabad. This study was carried out from 1 July 2019 to 31 December 2019. This study involved 238 patients of both genders aged between 12 and 70 years suspected of acute appendicitis on physical and ultrasound findings but with a normal TLC (4,500-11,000 WBCs/µL). The outcome variable was the frequency of acute appendicitis among such patients which was diagnosed upon surgery (inflamed appendix with free fluid) and histopathology of excised tissue (mucosal inflammation, neutrophil infiltrates, wall necrosis). Frequency of acute appendicitis was compared across various age and gender groups. Written informed consent was obtained from every patient.

Results

The mean age of the patients was 27.4±15.5 years. Majority (n = 167, 70.2%) of the patients were aged ≤25 years, followed by 40 (16.8%) patients aged ≥46 years and 31 (13.0%) patients aged between 26 and 45 years. There were 135 (56.7%) male and 103 (43.3%) female patients with a male to female ratio of 1.3:1. All of the patients (100.0%) had pain in the right iliac fossa (RIF) while rebound tenderness, anorexia, nausea/vomiting, fever and dysuria were noted in 83.6%, 79.0%, 73.9%, 63.9% and 15.1% patients, respectively. The diagnosis of acute appendicitis was made in 198 (83.2%) patients with normal TLC and suspicion of acute appendicitis on physical findings and ultrasound. When stratified, there was no statistically significant difference in the frequency of acute appendicitis across various age (p-value = 0.988) and gender (p-value = 0.913) groups.

Conclusion

In the present study, contrary to the routine impression that normal TLC rules out the differential diagnosis of acute appendicitis, a substantial proportion of patients with clinical and ultrasound suspicion of acute appendicitis but normal TLC had acute appendicitis which is worrisome as a missed case may later present with complications. The present study thus warrants cautious evaluation of clinically suspected cases with normal TLC count to avoid a missed appendicitis and improve the outcome in future surgical practice.

## Introduction

Acute appendicitis (AA) is the most common abdominal surgical emergency [1]. Patients present often with typical symptoms and physical findings. But sometimes, atypical presentation can occur. Diagnosis of acute appendicitis is clinical. Missed or delayed diagnosis of AA can lead to perforation of the appendix and significant morbidity [2,3]. Abdominal pain is the primary presenting complaint in most patients. According to epidemiological studies, 10% of the patients present with abdominal pain [4]. The colicky periumbilical pain followed by nausea/vomiting and the migration of pain to the right iliac fossa (RIF) is the classical sequence of symptoms. It was first described by Murphy but it may manifest in only 50% of the patients [5]. For Acute appendicitis, the lifetime risk among females and males is 6.7% and 8.6%, respectively [6].

Imaging techniques and Alvarado scoring can support the clinical diagnosis of appendicitis and decrease the negative appendectomies. Elevated total leukocyte count (TLC) supports the diagnosis of AA but a normal TLC does not necessarily rule out acute appendicitis [7]. For the diagnosis of AA, TLC is neither sensitive nor specific. It is due to the fact that high TLC can occur in more than 70% of etiologies causing right lower quadrant abdominal pain [8]. Most studies do show an association between elevated TLC and AA but its importance varies [9,10].

There is inconsistent information regarding the significance of TLC in the diagnosis of AA in patients presenting with RIF pain or with other symptoms of acute appendicitis. But a recent meta-analysis has shown that combined diagnostic accuracy of laboratory findings and clinical findings is very high [11]. According to a study, negative appendectomy rate was 19% in patients while 80.9% of patients had acute appendicitis who presented with normal TLC with suspicion of Acute Appendicitis [7]. According to another study, in patients with normal TLC, 6% of patients had no inflammation per-operatively, 12% had minimal inflammation and 82% had moderate to severe inflammation [12].

The aim of this study is to evaluate the frequency of acute appendicitis in patients with normal TLC. And apply the results to avoid negative appendectomy rates in patients presenting with RIF pain.

## Materials and methods

This descriptive cross-sectional study was conducted at the Department of Surgery, KRL Hospital Islamabad. It was carried out from 1 July 2019 to 31 December 2019. Sampling technique used was non-probability, consecutive sampling.

Sample size was calculated by WHO calculator taking confidence level at 95%. Anticipated population was 80.9% [7], absolute precision at 5%. The sample size calculated was 238.

Informed consent was taken from patients selected for study. Blood complete picture of the patients presenting with physical findings showing suspicion of AA was sent. Ultrasonography was done. Patients having age between 12 and 70 years, normal TLC count, having a suspicion of AA. USG Findings showing the presence of blind non-peristaltic loop in RIF, free fluid In RIF or peri appendiceal fat stranding & physical findings of pain RIF presenting within three days, nausea/vomiting, dysuria, anorexia, fever were included in the study.

Patients having any hematologic diseases like lymphomas, leukemias and multiple myeloma, emergency cases with perforated appendix, having any malignancy or patients with high TLC count were excluded from the study.

Open appendectomies were done by consultant/ senior registrar or by trainees under direct supervision. Per-operative findings were noted and recorded. The specimen was sent to KRL hospital Laboratory for histopathology. Patient was called for follow-up after one week with histopathology report signed by a Consultant pathologist of KRL hospital.

All the collected data were entered and analyzed using SPSS Version 21.0 (IBM Corp., Armonk, NY). Qualitative variables like gender, pain RIF at presentation, nausea/vomiting, dysuria, anorexia, fever, rebound tenderness, confirmed diagnosis of AA on per-operative findings and histopathology have been presented as frequency and percentage. While quantitative variables like age have been presented as mean ±SD. Frequency of AA has been compared across various subgroups based on age and gender and chi-square test has been applied taking p-value≤0.05 as statistically significant.

## Results

The age of the patients ranged from 12 years to 70 years with a mean of 27.4±15.5 years. Majority (n = 167, 70.2%) of the patients were aged ≤25 years, followed by 40 (16.8%) patients aged ≥46 years and 31 (13.0%) patients aged between 26 and 45 years. There were 135 (56.7%) male and 103 (43.3%) female patients with a male to female ratio of 1.3:1. All of the patients (100.0%) had pain in the RIF while rebound tenderness, anorexia, nausea/vomiting, fever and dysuria were noted in 83.6%, 79.0%, 73.9%, 63.9% and 15.1% patients respectively. These findings have been summarized in Table 1.

**Table 1:** Baseline characteristics of the study population.

Characteristics	Participants, n = 238
Age (years)	27.4±15.5
≤25 years	167 (70.2%)
26-45 years	31 (13.0%)
≥46 years	40 (16.8%)
Gender	
Male	135 (56.7%)
Female	103 (43.3%)
Physical findings	
Pain in right iliac fossa	238 (100.0%)
Rebound tenderness	199 (83.6%)
Anorexia	188 (79.0%)
Nausea/vomiting	176 (73.9%)
Fever	152 (63.9%)
Dysuria	36 (15.1%)

The diagnosis of AA was made in 198 (83.2%) patients with normal TLC and suspicion of AA on physical findings and ultrasound as shown in Table 2

**Table 2:** Frequency of acute appendicitis on per-operative and histopathological findings in patients with normal TLC. TLC: total leukocyte count.

Acute appendicitis	Frequency (n)	Percent (%)
Yes	198	83.2%
No	40	16.8%
Total	238	100.0%

When stratified, there was no statistically significant difference in the frequency of AA across various age (p-value = 0.988) and gender (p-value = 0.913) groups as shown in Table 3.

**Table 3:** Comparison of acute appendicitis across various subgroups of patients with normal TLC. TLC: total leukocyte count.

Subgroups	n	Acute appendicitis, n (%)	p-value
Age (years)			
≤25 years	167	139 (83.2%)	0.988
26-45 years	31	26 (83.9%)	
≥46 years	40	33 (82.5%)	
Gender			
Male	135	112 (83.0%)	0.913
Female	103	86 (83.5%)	

Chi-square test, observed difference was statistically insignificant.

## Discussion

AA is the most common cause of the acute abdomen requiring surgery with a lifetime risk of 7%, which is maximal in childhood and declines steadily with age as the lymphoid tissue and vascularity atrophy [1]. Although only a few patients progress to the potentially lethal complications, early surgery for all patients with suspected appendicitis has become the definitive method of preventing severe peritoneal sepsis [2]. Recent advances in interventional radiological techniques for peritonitis have significantly reduced the morbidity and mortality of physiologically severe complicated abdominal infections. CT scan is the current gold standard yet, the diagnosis of acute appendicitis is usually made on various clinical findings [2]. However, a missed case of acute appendicitis is a catastrophe as it later presents with life-threatening complications and results in medicolegal issues [1,2]. Raised total leukocyte count is a frequent finding in patients with acute appendicitis. As a convention, a normal leukocyte count usually rules out the differential diagnosis of acute appendicitis [3]. Recent studies claimed that a substantial proportion of patients with normal TLC had acute appendicitis and warranted a careful evaluation of such cases before sending them home [7,12,13,14]. However, the reported frequency of acute appendicitis among TLC normal patients varied greatly among studies which necessitated the present study.

In the present study, the mean age of the patients was 27.4±15.5 years. Majority (n = 167, 70.2%) of the patients were aged ≤25 years, followed by 40 (16.8%) patients aged ≥ 46 years and 31 (13.0%) patients aged between 26-45 years. A similar mean age among patients of acute appendicitis was previously reported [15] in 2013 (26±11 years) in India and Al-Shahwany et al [16] in 2012 (27±12 years) in Iraq. Kanumba et al [17] in 2011 observed a similar mean age of 29.64±12.97 years among acute appendicitis patients in Africa. Tsai et al [18] in 2015 reported much higher mean age of 33.0±22.0 years in Taiwan. A relatively lower mean age of 24.80±9 years was observed by Memon et al [19] In 2009 in patients presenting at Pakistan Institute of Medical Sciences Islamabad with right iliac fossa pain. Jalil et al [20] in 2011 (22.27±7.67 years) and Soomro et al [21] in 2008 (20.47 years) observed much lower mean age in local population. A similar higher proportion of younger age group among such patients was also observed by Talukder et al [22] in 2009 who observed that 71% of the patients with acute appendicitis were aged between 10 and 30 years.

We observed that there were 135 (56.7%) male and 103 (43.3%) female patients with a male to female ratio of 1.3:1. A similar male predominance among such patients has also been observed by Jalil et al [20] in 2011 (58% vs. 42%), Soomro et al [21] in 2008 (66.07% vs. 33.92%), Memon et al [19] in 2009 (65% vs. 35%) and Memon et al [23] in 2013 (71.8% vs. 28.2%) in local population. Talukder et al [22] (58% vs. 42%) in the Bangladeshi population, Beek et al [24] in 2015 (53% vs. 47%) in the Netherlands and Pogorelić et al [25] in 2015 (55.3% vs. 44.7%) in European population also reported a similar male predominance among such patients. Kanumba et al [17] however observed female predominance (29.1% vs. 70.9%) in African patients of acute appendicitis.

In the present study, all of the patients (100.0%) had pain in RIF while rebound tenderness, anorexia, nausea/vomiting, fever and dysuria were noted in 83.6%, 79.0%, 73.9%, 63.9% and 15.1% patients, respectively. Similar frequency of RIF pain, rebound tenderness, anorexia, nausea/vomiting and fever has also been observed by Dholia et al [26] (2010) at Chandka Medical College Hospital Larkana who reported it to be 100.0%, 75.0%, 82.0%, 70.0% and 67.0%, respectively. In a recent similar study in Nepal, Rabindra et al [27] (2018) reported a similar frequency of RIF pain (100.0%), rebound tenderness (82.1%), anorexia (80.2%) and nausea/vomiting (74.5%) in patients with clinically suspected acute appendicitis.

The diagnosis of acute appendicitis was made in 198 (83.2%) patients with normal TLC and suspicion of acute appendicitis on physical findings and ultrasound. When stratified, there was no statistically significant difference in the frequency of acute appendicitis across various age (p-value = 0.988) and gender (p-value = 0.913) groups.

Our observation matches with that of Alam et al [13] (2014) who reported a similar frequency of 83.9% for acute appendicitis in patients with clinical suspicion but normal TLC count at Pakistan Institute of Medical Sciences, Islamabad. In another local study, Jamaluddin et al [28] (2013) reported this frequency to be 82.0% among patients presenting at Dow University of Health Sciences with clinical suspicion of acute appendicitis but with normal total leukocyte count. Sadettin Er et al [7] (2018) reported a similar frequency of acute appendicitis (80.9%) among TLC normal patients in Turkey. Singh et al [14] (2016) reported a slightly higher frequency of 88.5% in India.

A study in Iran shows that increased leukocyte count, even within the normal range can be associated with chronic complications in type 2 diabetes. Accompanied by other markers, chronic inflammation that can be shown by this factor could be related to pathogenesis and the progression of these diabetes-related complications [29].

The present study adds to the already published local and international research evidence on the topic. The strengths of the present study were its large sample size of 238 cases. We also stratified the data to address effect modifiers. In the present study, contrary to the routine impression that normal TLC rules out the differential diagnosis of acute appendicitis, a substantial proportion of patients with clinical and ultrasound suspicion of acute appendicitis but normal TLC actually had the disease. This observation is in line with the previous reports in this regard and is worrisome as a missed case may later present with complications. The present study thus warrants cautious evaluation of clinically suspected cases with normal TLC count in the form of a CT scan abdomen and admission with repeated intermittent evaluation instead of sending the patient home to avoid a missed appendicitis and improve the outcome in future surgical practice.

A very strong limitation to the present study was that inflammatory response with elevation of TLC count takes time to develop and that might be a reason for acute appendicitis with normal TLC. We didn’t stratify the data for time since the onset of symptoms which might have identified it as a probable confounding factor. A study addressing this limitation is necessary and is highly recommended in future research.

## Conclusions

In the present study, contrary to the routine impression that normal TLC rules out the differential diagnosis of acute appendicitis, a substantial proportion of patients with clinical and ultrasound suspicion of acute appendicitis but normal total leukocyte count had acute appendicitis which is worrisome as a missed case may later present with complications. The present study thus warrants cautious evaluation of clinically suspected cases with normal TLC to avoid a missed appendicitis and improve the outcome in future surgical practice.
